# Supplementary material for: Cytoplasmic forces functionally reorganize nuclear condensates in oocytes
Source: Nat Commun. 2022 Aug 29;13:5070. doi: 10.1038/s41467-022-32675-5 (PMC9424315; doi:10.1038/s41467-022-32675-5)
Supplement: Supplementary file 1 — Supplementary Information File [file 41467_2022_32675_MOESM1_ESM.pdf]

Supplementary Information for

Cytoplasmic forces functionally reorganize nuclear condensates in oocytes

Adel Al Jord, Gaëlle Letort, Soline Chanet, Feng-Ching Tsai, Christophe Antoniewski, Adrien Eichmuller, Christelle Da Silva, Jean-René Huynh, Nir S. Gov, Raphaël Voituriez, Marie-Émilie Terret and Marie-Hélène Verlhac

Correspondence to: adel.aljord@college-de-france.fr

This PDF file includes:
Supplementary Figures 1 to 10
Legends for Supplementary Figures 1 to 10
Legends for Supplementary Movies 1 to 7
Legends for Supplementary Data 1 to 5
Supplementary references

Other Supplementary Materials include:
Supplementary Movies 1 to 7 and Supplementary Data 1 to 5

Supplementary Fig. 1

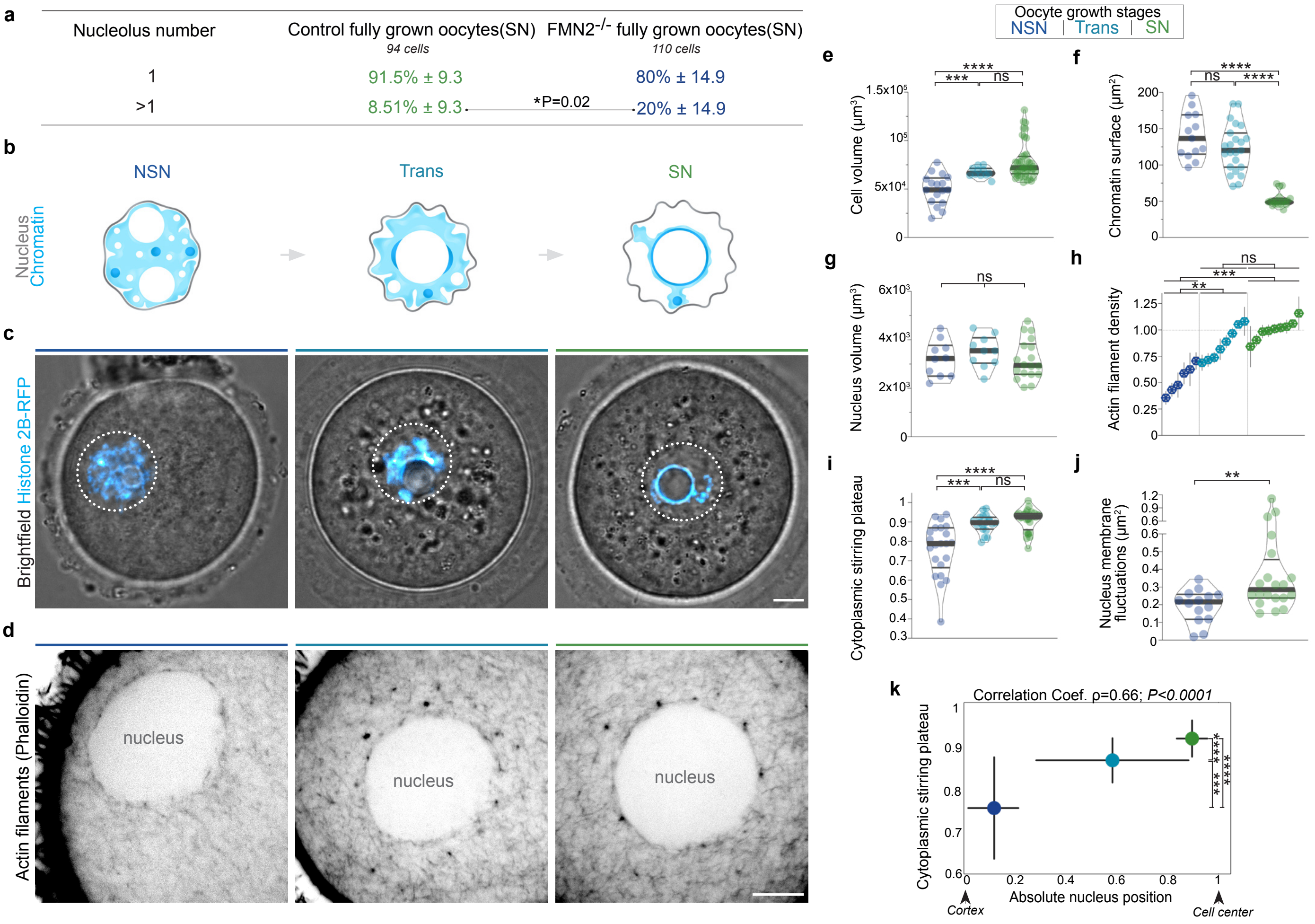

Supplementary Fig. 1 | Nucleolus number in Control and F-actin mutant fully grown mouse oocytes and cytoplasmic random stirring relative to oocyte growth stages

(**a**) Nucleolus number quantification showing a ~2.35-fold enrichment in the proportion of cells presenting more than one nucleolus in fully-grown SN FMN2-/- oocytes when compared to SN Controls. (**b**) Illustration of chromatin configuration evolution (NSN-Trans-SN see ([1–3])) coinciding with oocyte growth subcategories (mid-antral, late-antral, and fully grown). The nuclear membrane (gray) is depicted according to its fluctuation intensity at distinct stages of growth (also see (4) and (**j**)). (**c**) Merge of bright-field and fluorescent labeling of live control oocytes expressing the chromatin marker H2B-RFP at distinct growth stages (0.5μm z-planes), linking chromatin configuration and nucleus position evolution with mouse oocyte growth progression; nucleus region is outlined in white. (**d**) Actin filaments (F-actin) stained with Phalloidin relative to oocyte growth progression; note the increase of cytoplasmic F-actin network density as of the Trans stage coinciding with nucleus centering; F-actin is absent from the nucleus interior, similar to what can be observed in human oocytes[5]. (**e**) Quantifications of cell volume in mid-antral (NSN), late-antral (Trans), and fully grown (SN) fixed oocytes; cell number, NSN=17, Trans=11, SN=43. (**f**) Chromatin surface relative to oocyte growth; measurements were made on 40μm z-projections of fixed cells; cell number, NSN=13, Trans=23, SN=18. (**g**) Nucleus volume quantifications relative to growth stages in fixed oocytes; cell number, NSN=10, Trans=10, SN=18. (**h**) Quantifications of cytoplasmic F-actin density per cell relative to oocyte growth stages; 20 measurements of Phalloidin-stained F-actin per cell represented by mean±s.d. spanning different z-planes from top to bottom of each cell and values normalized to the mean SN density value; distribution medians used to compare stages statistically. (**i**) Cytoplasmic stirring intensity plateaus relative to growth stage obtained with image correlation analyses of cytoplasmic stirring in live oocytes; cell number, Control NSN=22, Trans=19, SN=28. (**j**) Nucleus membrane fluctuations in live NSN and SN oocytes measured as in ([4]); cell number, NSN=14, SN=20. (**k**) Cytoplasmic stirring intensity plateaus correlated to absolute nucleus positions in live growing oocytes; absolute nucleus position corresponds to 1- ρ, with ρ quantified as in ([6]); cell number, NSN=16, Trans=16, SN=20; error represents mean±s.d., Spearman correlation coefficient ρ=0.66, ****P<0.0001. Violin plots with median±quartiles; P values derived from Fisher's exact test of proportions (**a**), two-tailed Mann-Whitney U-Tests (**e, f, g, i, j,** and **k** for nucleus position tests), and Kruskal-Wallis Test (**h**), ns, not significant, P>0.0735, **P<0.0068, ***P<0.0006, ****P<0.0001. Oocyte growth color codes based on cytoplasmic stirring intensities; scale bars, 5μm. Source data are provided as a Source Data file.

Supplementary Fig. 2

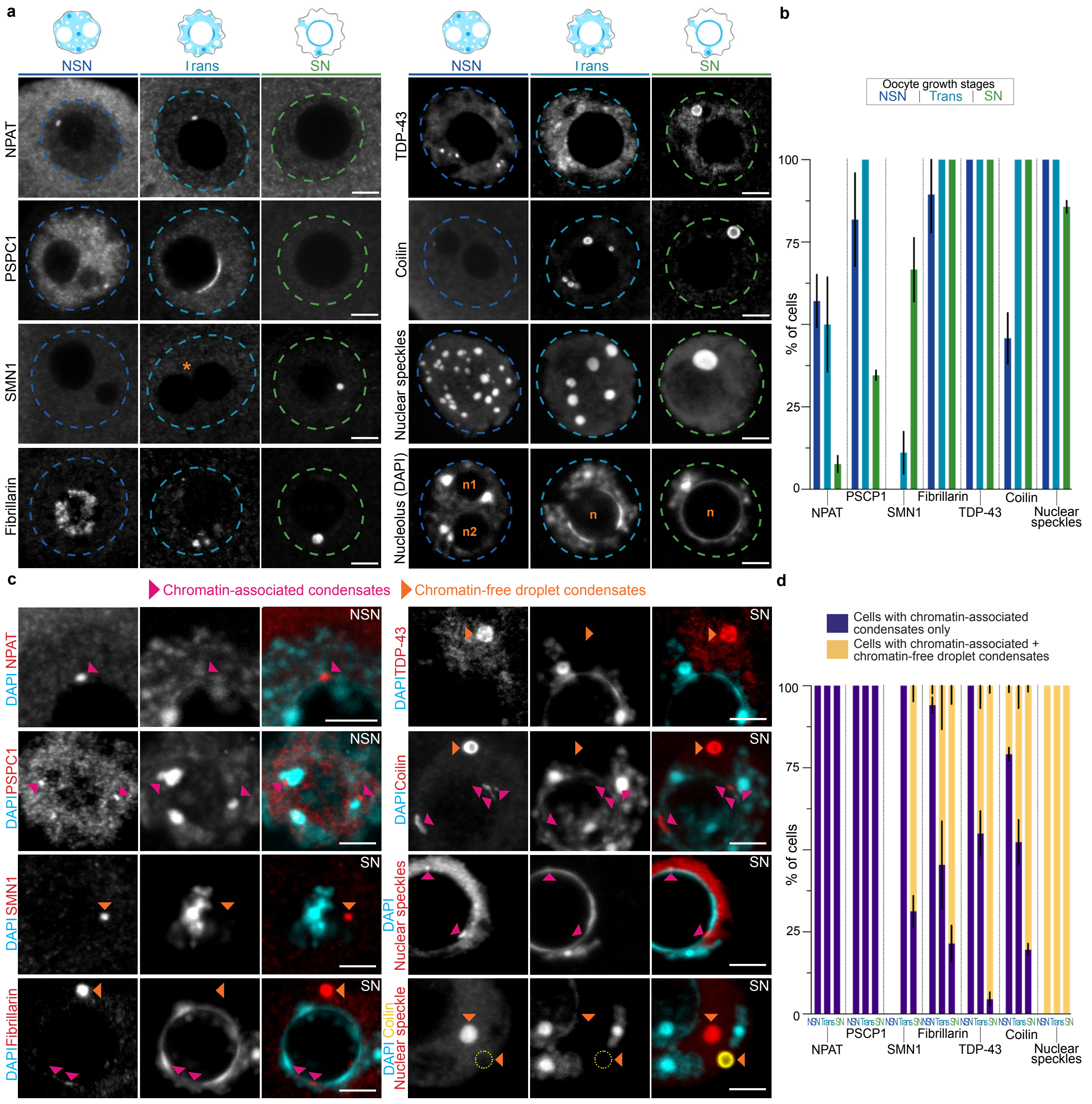

Supplementary Fig. 2 | Immunocytochemistry screen of diverse nuclear biomolecular condensates in growing oocytes with classification into chromatin-associated and chromatin-free (droplet) condensates

(**a**) Representative immunostainings of screened nuclear biomolecular condensates using known immunomarkers[7,8] relative to oocyte growth stages; NPAT (Nuclear Protein, Coactivator of Histone Transcription) is a marker of Histone Locus Bodies, PSPC1 (Paraspeckle Component 1) of paraspeckles, SMN1 (Survival of Motor Neuron 1) of gems and rarely associated with Cajal bodies, Fibrillarin of nucleoli and rarely Cajal bodies, TDP-43 (TARDBP; TAR DNA-Binding Protein 43) of paraspeckles, of TDP-43 bodies and of Cajal bodies, Coilin of Cajal bodies, and nuclear speckles stained with Abcam ab11826 antibody (SC35/SRSF2); Fibrillarin is notably nucleolar in NSN cells before becoming mostly nucleoplasmic as of the Trans stage suggesting the restructuring of the multiphase nucleolus[9]; due to this nucleolar phase restructuring observed as of the Trans stage, the nucleolus (marked with the letter "n") loses Fibrillarin positivity and becomes recognizable only by signal exclusion and the surrounding DNA (stained with DAPI); asterisk in the SMN1 row indicates a visible nucleolar fusion; single 0.5µm z-planes are shown except for nuclear speckles which are 20µm z-projections; nucleus regions are outlined with dashed lines color coded relative to cytoplasmic stirring intensities at different growth stages. (**b**) Proportions of NSN, Trans, and SN oocytes that contain tested nuclear biomolecular condensates in their nucleus; cell number, NPAT NSN=14, Trans=6, SN=13, PSPC1 NSN=11, Trans=8, SN=26, SMN1 NSN=16, Trans=9, SN=24, Fibrillarin NSN=19, Trans=11, SN=14, TDP-43 NSN=9, Trans=10, SN=22, Coilin NSN=24, Trans=22, SN=56, nuclear speckles NSN=37, Trans=24, SN=42. (**c**) Images from the immunocytochemistry screen showing the two subpopulations of nuclear biomolecular condensates observed at various stages of oocyte growth; smaller, irregularly shaped chromatin-associated condensates indicated by fuchsia arrowheads and larger, round chromatin-free droplet condensates indicated by orange arrowheads; DNA is stained with DAPI; all images are z-projections, 3µm for NPAT and PSPC1, 2µm for SMN1, 5µm for Fibrillarin and bottom SRSF2, 10µm for TDP-43 and Coilin, and 1µm for top nuclear speckles; note that in the bottom nuclear speckles merge, a Coilin droplet is shown in yellow; note also that the nucleolus is an exception to the binary rule of classification since it always is a droplet that is in tight contact with and surrounded by chromatin. (**d**) Proportions of NSN, Trans, and SN oocytes containing nuclear condensates that are exclusively chromatin-associated (purple) or containing both chromatin-associated condensates and droplets (yellow); tested condensate immunomarkers and growth stages are indicated below the histogram; cell number, NPAT NSN=8, Trans=3, SN=1, PSPC1 NSN=9, Trans=8, SN=9, SMN1 NSN=0, Trans=1, SN=16, Fibrillarin NSN=17, Trans=11, SN=14, TDP-43 NSN=5, Trans=20, SN=22, Coilin NSN=11, Trans=21, SN=56, nuclear speckles NSN=37, Trans=24, SN=42. Bars above images are color coded relative to cytoplasmic stirring intensities at corresponding growth stages; scale bars, 5µm.Source data are provided as a Source Data file.

Supplementary Fig. 3

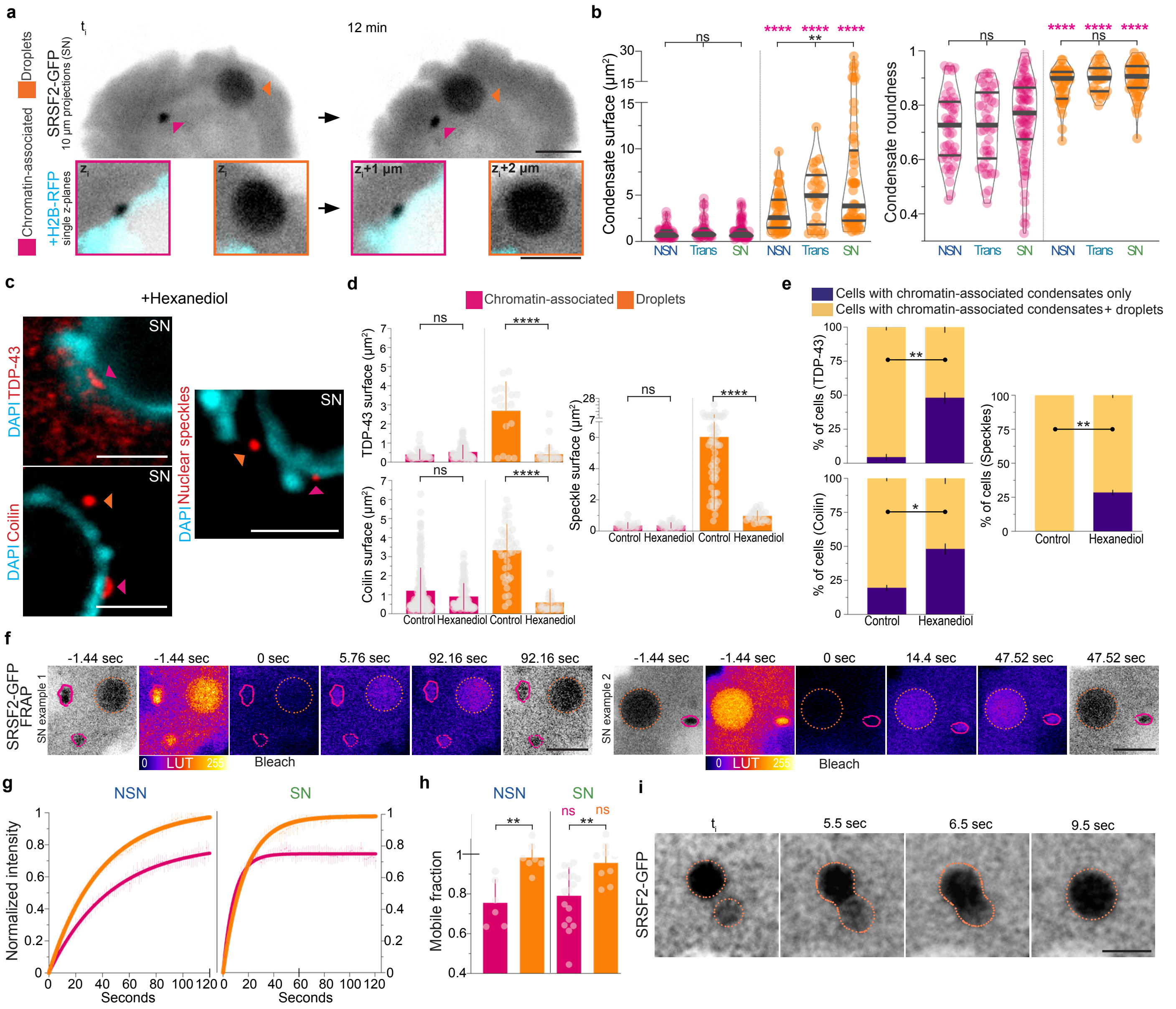

Supplementary Fig. 3 | Droplet condensates behave like liquids whereas chromatin-associated condensates do not

(**a-b**) Condensate subtype mobility, size and shape. (a) Time-lapse frames showing high spatiotemporal mobility of a large SRSF2-GFP droplet (orange) and low mobility of a small chromatin-associated condensate (fuchsia) that typically remains stably bound to chromatin (H2B-RFP) in a live oocyte. (**b**) Quantifications of surface and roundness of SRSF2-GFP chromatin-associated or droplet condensates in live oocytes at all stages of growth. Droplets are significantly bigger and rounder at all stages of growth; condensate number, chromatin-bound NSN=49, Trans=46, SN=82, droplets NSN=49, Trans=28, SN=58. (**c-e**) Treatment with 1-6-hexanediol, a disruptor of weak hydrophobic molecular interactions that are key for liquid-like behavior[10–12], dissolves droplets without affecting chromatin-bound condensates. (**c**) Representative immunostainings of TDP-43 (0.5µm z-plane) and Coilin (5µm z-projection) in SN oocytes treated with 1-6-hexanediol [1%] for 7 minutes (TDP-43 and Coilin) or [5%] for 10 minutes (nuclear speckles), and showing Control-like chromatin-associated condensates (fuchsia arrowheads) and small droplets (orange arrowheads); DNA stained with DAPI (in blue). (**d**) Quantifications of TDP-43, Colin, and nuclear speckle condensate surfaces in SN oocytes following 1-6-hexanediol [1% or 5%] treatments and showing a size-decrease only for droplets; condensate number, TDP-43 chromatin-associated|droplets, Control=38|18, Hexanediol=84|29, Coilin chromatin-associated|droplets, Control=183|44, Hexanediol=108|29, nuclear speckles chromatin-associated|droplets, Control=22|62, Hexanediol=26|25. (**e**) Proportion of SN oocytes containing TDP-43 and Coilin condensates that are exclusively chromatin-associated (purple) or containing both chromatin-associated condensates and droplets (yellow); cell number, TDP-43 Control=22, Hexanediol=27, Coilin Control=56, Hexanediol=27, nuclear speckles Control=42, Hexanediol=14. (**f-h**) High fluorescence recovery of SRSF2-GFP droplets in NSN and SN oocytes. (**f**) Representative FRAP sequences of SRSF2-GFP chromatin-associated condensates (fuchsia) and droplets (orange). (**g**) Normalized fluorescence intensity recovery curves (mean±s.e.m.) with simple exponential fits of SRSF2-GFP chromatin-associated condensates and droplets in (left) NSN or (right) SN oocytes. (**h**) Mobile fractions of total populations for SRSF2-GFP relative to condensate sub-type and oocyte growth stage; mobile fractions correspond to recovery fractions evaluated at 120 seconds for NSN condensates and 60 seconds for SN condensates; condensate number, chromatin-associated NSN=6, SN=18, droplets NSN=7, SN=9. (**i**) Time-lapse images showing rapid SRSF2-GFP droplet coalescence in a live SN oocyte. Violin plots with median±quartiles (b), error bars represent mean±s.d. (**d** and **h**) and mean±s.e.m. (**e**); P values derived from two-tailed Mann-Whitney U-Tests (**b, d,** and **h**), Kruskal-Wallis Tests (**b**), or Fisher's exact test of proportions (**e**), ns, not significant, P>0.0648, *P=0.0102, **P<0.0054, ****P<0.0001; scale bars, 5µm.Source data are provided as a Source Data file.

Supplementary Fig. 4

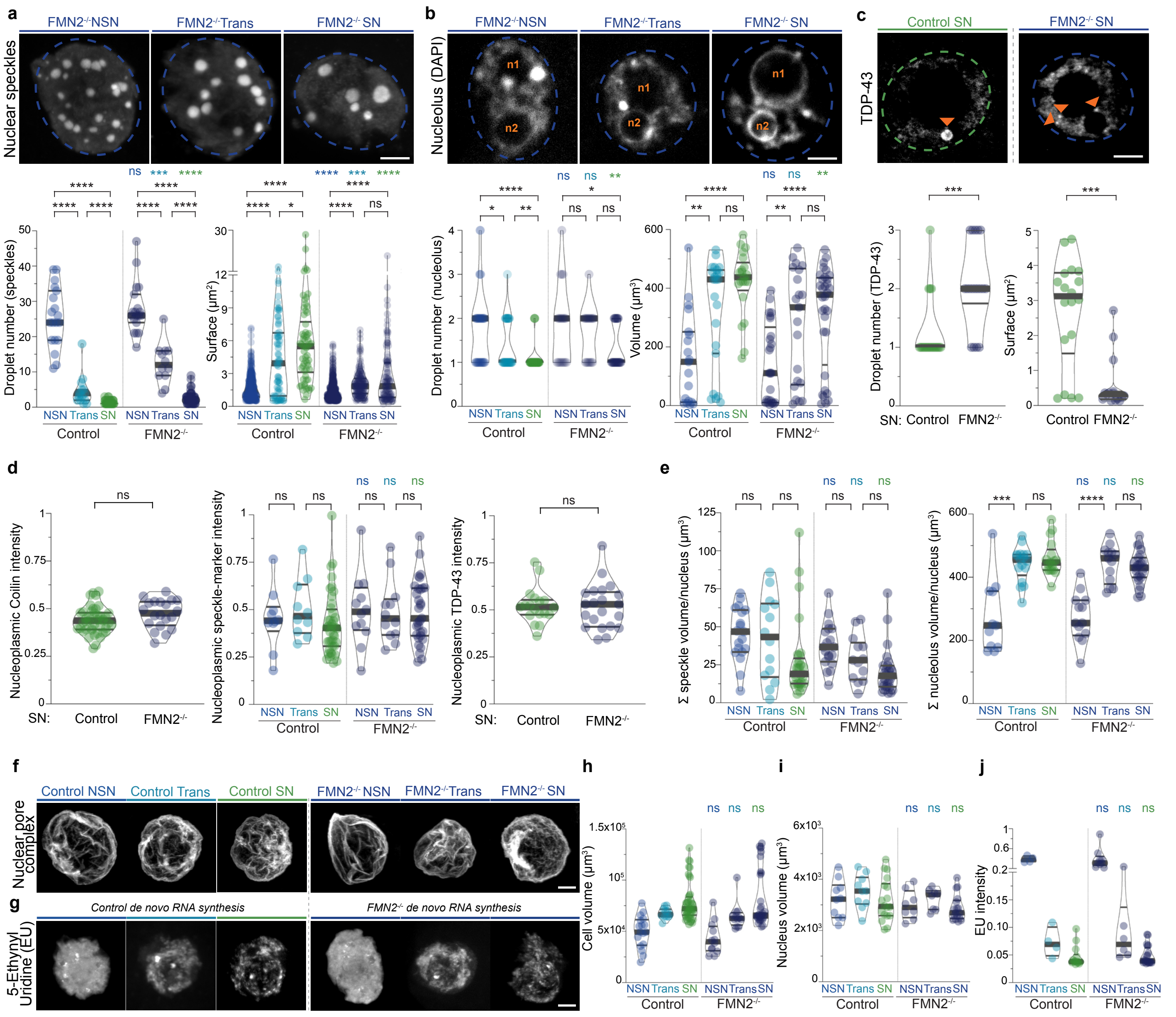

Supplementary Fig. 4 | Nuclear droplets are smaller and more abundant in growing F-actin mutant oocytes with disrupted cytoplasmic forces, but total condensate marker amounts, cell or nucleus volumes, and neo-transcription are comparable to Controls

(**a**) Immunostainings of nuclear speckles in growing F-actin mutant (FMN2-/-) oocytes and quantifications of droplet numbers and sizes in Control and FMN2-/- NSN, Trans, and SN oocytes (related to Fig.1d-e, also see Supplementary Fig.2a for Controls); cell number (left graph), Control NSN=19 ,Trans=15 ,SN=41, FMN2-/- NSN=19, Trans=12, SN=52; condensate number (right graph), Control NSN=450, Trans=69, SN=62, FMN2-/- NSN=528, Trans=149, SN=122. (**b**) Images showing nucleoli and DNA (DAPI) in growing FMN2-/- oocytes; nucleoli visible by signal exclusion and are marked with the letter "n" (see Supplementary Figs. 1a and 2a for Controls); graphs are quantifications of nucleolus numbers and volumes; cell number (left graph), Control NSN=38, Trans=42, SN=66; FMN2-/- NSN=19, Trans=17, SN=36; nucleoli number (right graph), Control NSN=19, Trans=23, SN=21, FMN2-/- NSN=25, Trans=21, SN=32. (**c**) Immunostainings of TDP-43 and quantifications of droplet numbers and sizes in SN Control and FMN2-/- oocytes; orange arrowheads indicate droplets; cell number (left graph), Control=20, FMN2-/-=18, condensate number (right graph), Control=18, FMN2-/-=15. (**d**) Total nucleoplasmic intensities for Coilin (left), nuclear speckles (center) and TDP-43 (right) in Control and FMN2-/- SN oocytes; cell number for Coilin, Control=43, FMN2-/-=20; cell number for nuclear speckles, Control NSN=10, Trans=10, SN=40, FMN2-/- NSN=12, Trans=12, SN=31; cell number for TDP-43, Control=18, FMN2-/-=21.(**e**) Sum of nuclear speckle (left) or nucleolus (right) volumes per nucleus in Control and FMN2-/- SN oocytes; cell number for nuclear speckles, Control NSN=19, Trans=14, SN=29, FMN2-/- NSN=19, Trans=13, SN=27; Nucleolus, Control NSN=11, Trans=17, SN=17, FMN2-/- NSN=13, Trans=13, SN=23. (**f**) Representative images of the nucleus membrane immunostained with the Mab414 antibody recognizing the nuclear pore complex in growing Control or FMN2-/- oocytes (top; 20-24µm z-projections). (g) Incorporation profiles of 5-Ethynyl Uridine[13] (EU, 1mM incubated for 240 minutes) that reflect de novo RNA synthesis in growing Control or FMN2-/- oocytes; 20µm z-projections show comparable drops in transcription with growth progression. (**h-j**) Quantifications of cell volume (**h**), nucleus volume (**i**), and EU signal intensity (**j**) in growing Control or FMN2-/- oocytes; cell number, (**h**) Controls same as in Supplementary Fig.1e, FMN2-/- NSN=12, Trans=12, SN=31, (**i**) Controls same as in Supplementary Fig.1g, FMN2-/- NSN=8, Trans=8, SN=17, (**j**) Controls NSN=4, Trans=4, SN=14, FMN2-/- NSN=9, Trans=16, SN=20. Nucleus regions in a-c outlined with dashed lines; violin plots with median±quartiles; P values derived from two-tailed Mann-Whitney U-Tests, ns, not significant, P>0.0559, *P<0.0319, **P<0.0097, ***P<0.0007, ****P<0.0001; colored "ns" and asterisks are statistical comparisons with Controls; oocyte growth color codes based on cytoplasmic stirring intensities; scale bars, 5µm.Source data are provided as a Source Data file.

Supplementary Fig. 5

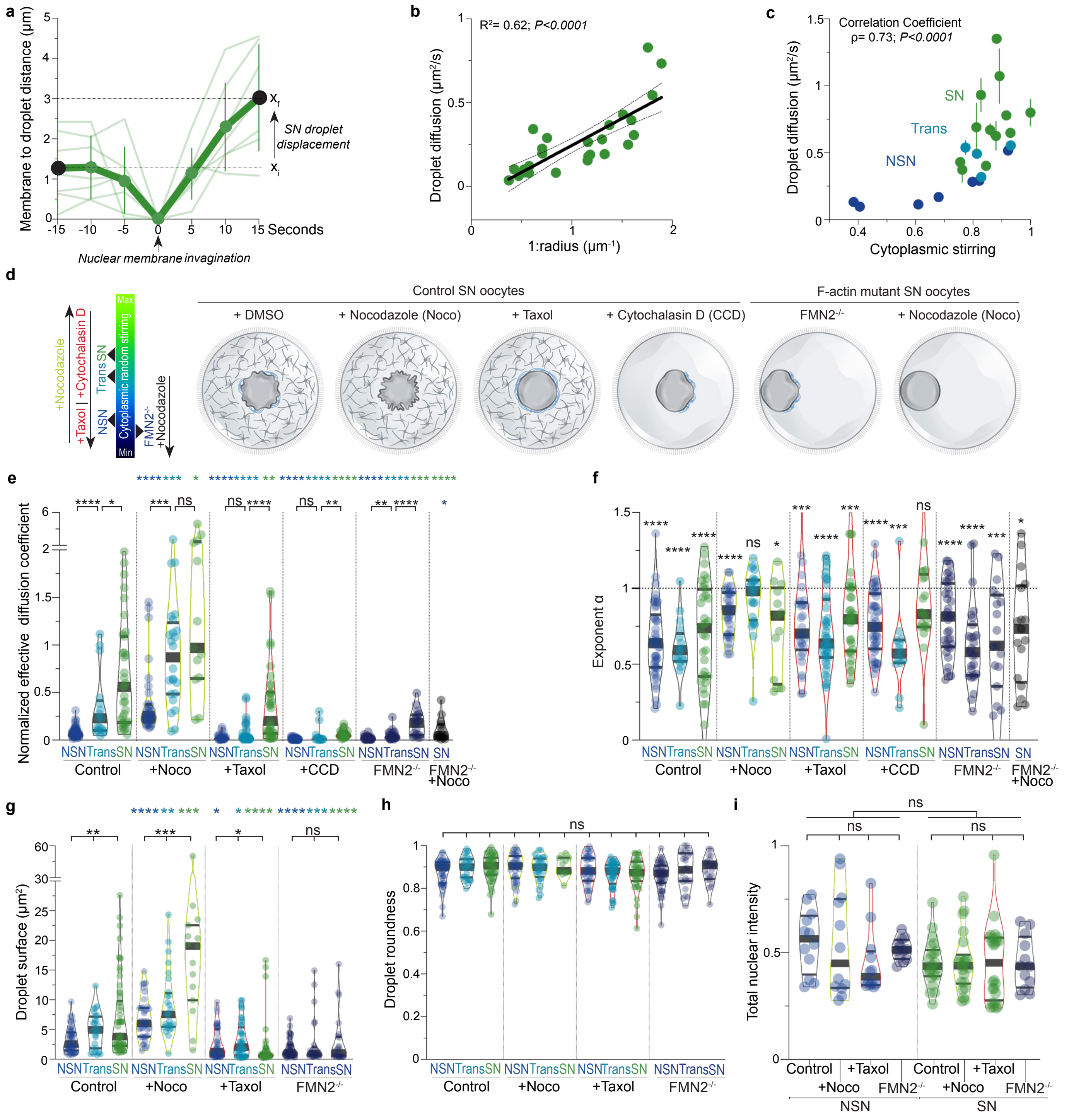

Supplementary Fig. 5 | Cytoplasmic forces boost diffusive dynamics of nuclear SRSF2-GFP droplets on second to minute timescales

(**a**) Cytoplasmic forces locally displace SRSF2-GFP droplets via nuclear membrane fluctuations; graph shows measurements of the decreasing distance between the nuclear membrane's invaginating tip and the SRSF2-GFP droplet that is pushed and displaced from x_i to x_f; mean±s.d. of 7 droplets in 6 SN oocytes from films similar to the ones in Fig.2a and Supplementary Movie 3. (**b**) Global SRSF2-GFP droplet diffusion inversely depends on droplet radius; 26 SN droplet diffusion coefficients plotted against the droplets' radii^{-1} with a simple linear regression±90%CI; the relation is consistent with the r^{-1} dependence expected from Stokes' law. (**c**) Global diffusion of nuclear SRSF2-GFP droplets correlates with the intensification of cytoplasmic stirring that occurs during oocyte growth; mean±s.e.m. of 23 droplet diffusion coefficients plotted against cytoplasmic stirring values obtained using image correlation analyses; Spearman correlation coefficient ρ is shown. (**d**) Illustration of tools used to modulate cytoplasmic forces and consequent nucleus agitation in growing oocytes4; Nocodazole amplifies nucleus agitation whereas Taxol obstructs it; Cytochalasin-D disrupts the actomyosin network and thus nucleus agitation; mutant FMN2-/- oocytes lack F-actin and only have residual microtubule-based agitation of the nucleus that is further diminished with the addition of Nocodazole; SN oocytes with actomyosin networks and microtubule organizing centers surrounding the nucleus are depicted when present, cytoplasmic microtubules are not illustrated. (e-f) Normalized effective diffusion coefficients of SRSF2-GFP droplets (**e**) and diffusive exponents α (**f**) in growing Control oocytes, oocytes incubated with Nocodazole, Taxol, or Cytochalasin-D, FMN2-/- oocytes, and FMN2-/- oocytes incubated with Nocodazole; effective diffusion coefficients were normalized by droplet size (D_{eff} in $\mu m^2 s^{-1}$ x 3/2 πr in µm); droplet number, Control NSN=46, Trans=17, SN=35, Nocodazole NSN=32, Trans=24, SN=13, Taxol NSN=29, Trans=43, SN=36, Cytochalasin-D NSN=48, Trans=14, SN=21, FMN2-/- NSN=36, Trans=27, SN=19, FMN2-/-+Nocodazole SN=17. (**g-h**) Surface (**g**) and roundness (**h**) measurements of SRSF2-GFP droplets 3 hours post microinjection. Nocodazole and Taxol were added 2 hours post microinjection for an hour; droplet number, Control NSN=49, Trans=28, SN=58, Nocodazole NSN=38, Trans=28, SN=15, Taxol NSN=40, Trans=44, SN=39, FMN2-/- NSN=50, Trans=26, SN=24. (**i**) Total nucleoplasmic SRSF2-GFP intensity measured on 40µm z-projections in NSN and SN Control oocytes, oocytes incubated with Nocodazole or Taxol, and FMN2-/- oocytes; droplet number, Control NSN=12, SN=22, Nocodazole NSN=12, SN=22, Taxol NSN=12, SN=24, FMN2-/- NSN=12, SN=12. Violin plots with median±quartiles; colored asterisks are statistical comparisons with Controls; P values derived from two-tailed Mann-Whitney U-Tests (**e-g**), Wilcoxon Signed-Rank Test (**f**; compared with a hypothetical α=1 median), and Kruskal-Wallis Tests (**g-i**), ns, not significant, P>0.0701, *P<0.0478, **P<0.0054, ***P<0.001, ****P<0.0001; oocyte growth color codes based on cytoplasmic stirring intensities. Source data are provided as a Source Data file.

Supplementary Fig. 6

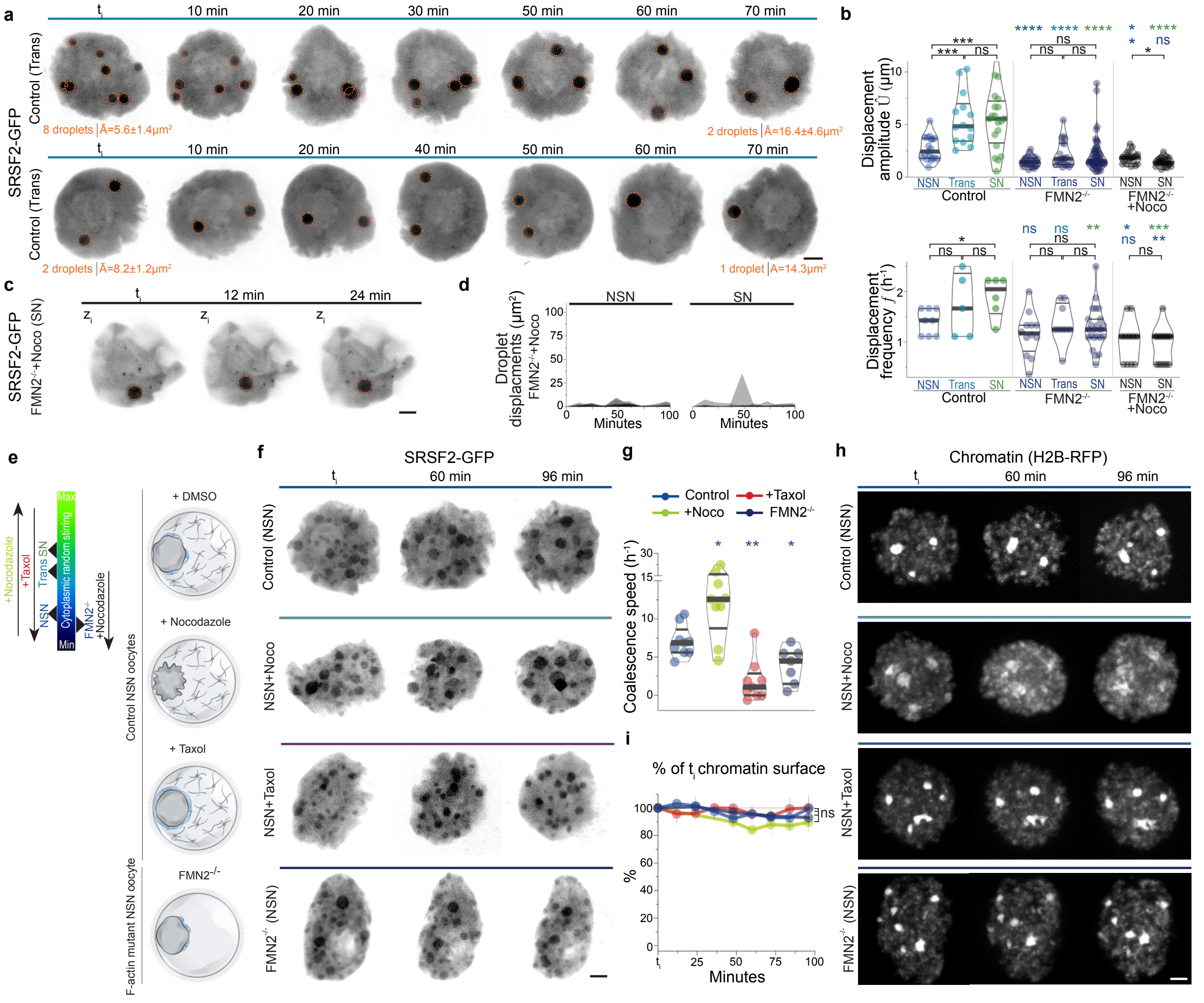

Supplementary Fig. 6 | Cytoplasmic forces drive stochastic displacements and collision-coalescence of nuclear SRSF2-GFP droplets on minute to hour timescales

(**a**) Additional examples of Control SRSF2-GFP droplet minute-scale 3D-dynamics (50µm z-projections) in Trans cells highlighting random nucleoplasmic displacements and collision-coalescence dynamics; droplet number and mean surface evolution indicated below in orange. (**b**) Quantifications of displacement amplitudes (top) and frequencies (bottom) obtained with 3D-tracking of random SRSF2-GFP droplet displacements in nuclei of growing Control, FMN2-/-, and FMN2-/-+Nocodazole oocytes; number of amplitude measurements, Control NSN=19, Trans=14, SN=20, FMN2-/- NSN=25, Trans=21, SN=57, FMN2-/-+Nocodazole NSN=20, SN=22; droplet number for frequency, Control NSN=8, Trans=5, SN=6, FMN2-/- NSN=12, Trans=9, SN=23, FMN2-/-+Nocodazole NSN=11, SN=14; related to Fig.2g-h. (**c**) Time-lapse (single 0.5µm z-plane) showing weak minute-timescale displacements of a tracked SRSF2-GFP droplet in the nucleoplasm of a fully-grown SN FMN2-/- oocyte incubated with Nocodazole. (**d**) 3D-tracks of SRSF2-GFP droplets in NSN and SN FMN2-/- oocytes incubated with Nocodazole; droplet displacement amplitude Û and frequency *f* in **b**; droplet number, NSN=11, SN=14. (**e**) Illustration of tools used to modulate cytoplasmic forces in NSN oocytes for SRSF2-GFP droplet collision-coalescence experiments in **f-h**, Fig.2j and Supplementary Movie 6; NSN oocytes with actomyosin networks and microtubule organizing centers surrounding the nucleus are depicted when present, cytoplasmic microtubules are not illustrated.(**f**) 3D-dynamics (40µm z-projections) of SRSF2-GFP droplets in NSN Control oocytes, oocytes incubated with Nocodazole or Taxol, and FMN2-/- oocytes; droplet number and size evolution quantified in Fig.2j; see Supplementary Movie 6. (**g**) Quantification of droplet coalescence speed of cells quantified in Fig.2j; Control n=9, Noco n=9, Taxol n=9, FMN2-/- n=7. (**h**) Time-lapse images of chromatin (H2B-RFP; 40µm z-projections) showing absence of chromatin condensation in NSN oocytes analyzed in **f** and Fig.2j. (**i**) Quantification of chromatin surface evolution in % of initial surface; oocyte number, 3-4 per condition. Violin plots with median±quartiles (**b, g**); error bars represent mean±s.e.m. (**i**); colored asterisks in **b** are statistical comparisons with Control droplets; P values derived from two-tailed Mann-Whitney U-Tests (**b, g**) and Wilcoxon Matched-Pairs Signed Rank Tests (**i**), ns, not significant, P>0.0625, *P<0.042, **P<0.0079, ***P<0.0008, ****P<0.0001; oocyte growth color codes based on cytoplasmic stirring intensities and inhibitor treatment; scale bars, 5µm. Source data are provided as a Source Data file.

Supplementary Fig. 7

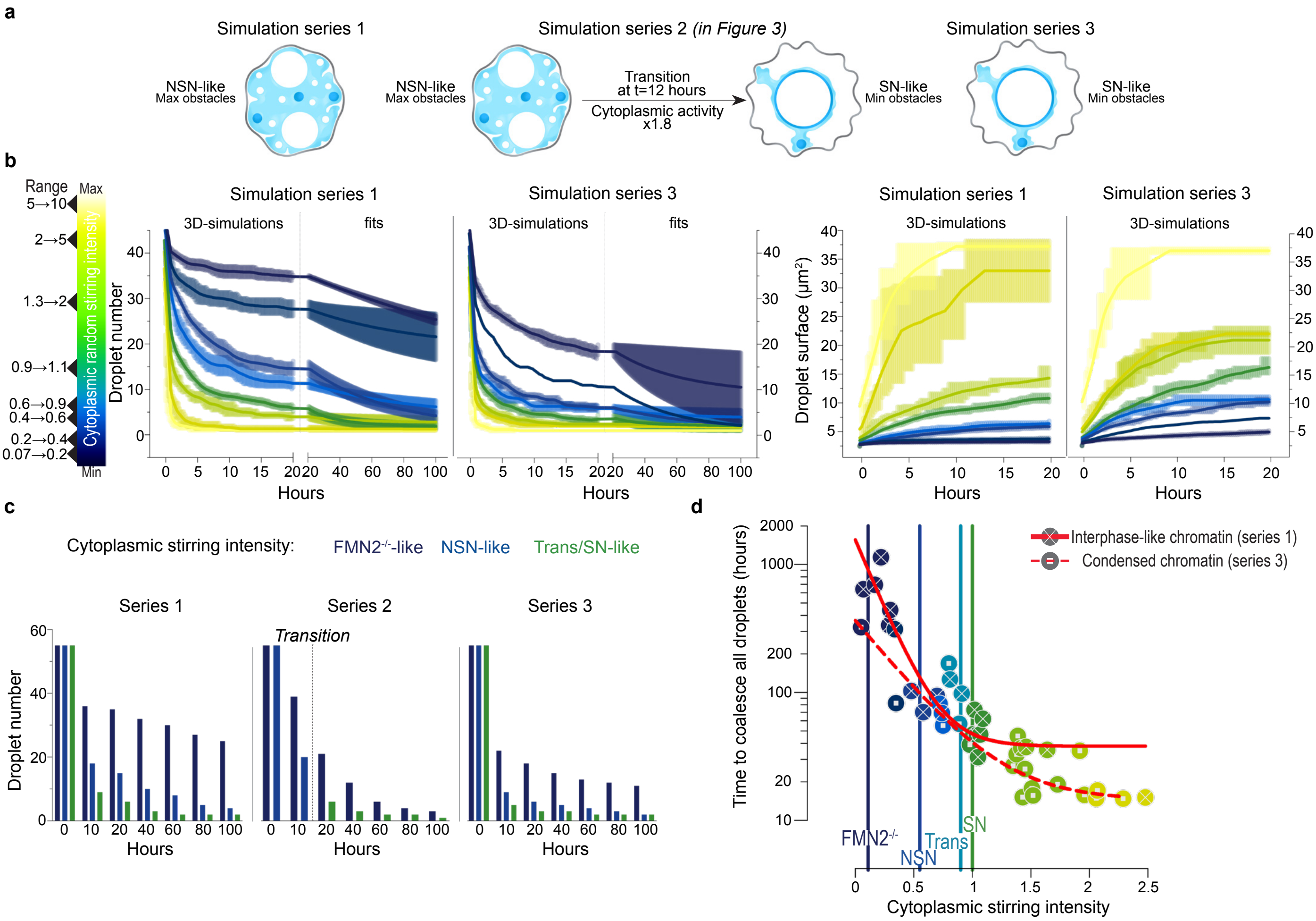

Supplementary Fig. 7 | 3D-simulations of nuclear speckle collision-coalescence dynamics relative to cytoplasmic forces on long timescales of several days

(**a**) Schematic representation of the simulation regimes tested; Series 1: NSN-like configuration with interphase chromatin-like obstacles widely spread in the nucleus and cytoplasmic stirring activity maintained constant (e.g. 0.55 for Control, 1.05 for Nocodazole, and 0.11 for FMN2-/-); Series 2: NSN-to-SN-like configuration; first 12 hours of simulations are performed with the same parameters as in NSN-like simulations of series 1; nuclear obstacle and cytoplasmic stirring activity switch occurs at 12 hours whereby 40 % of chromatin-like obstacles surround the nucleolus and cytoplasmic activity is nearly doubled to mimic the transition into the SN-like condition, physiologically marked by chromatin condensation and cytoplasmic force intensification; Series 3: SN-like configuration with 40 % of chromatin-like obstacles surrounding the nucleolus and cytoplasmic stirring activity maintained constant (e.g. 1 for Control, 1.9 for Nocodazole, and 0.2 for FMN2-/-). (**b**) Droplet number (left) and size (right) evolution in NSN-like (series 1) and SN-like (series 3) simulations relative to varying cytoplasmic stirring activities; simulations are binned by range of activities (color gradient), from lower values (dark blue) to higher values (light yellow); droplet diffusion and fusions are simulated for 20 hours with longer-term dynamics predicted by a decreasing exponential fit calculated on the last 5h of the simulations; simulation number, 1st series=44; 3rd series=41. (**c**) Comparative histograms of droplet number evolution from the three simulations with cytoplasmic stirring activity intensities selected to resemble that of NSN and SN Control and F-actin mutant oocytes; note that in series 2, blue NSN-like bars switch to green Trans/SN-like bars after the transition. (**d**) Time necessary to coalesce 45 droplets relative to cytoplasmic stirring intensity comparing series 1 (NSN-like nucleus state with interphase-like chromatin) and 3 (SN-like nucleus state with condensed chromatin); time in the y-axis is calculated from fits in **b**; one-phase decay fits are in red and intersect vertical colored bars showing the simulated stirring activities corresponding to experimental ones in Controls and F-actin mutants; comparing plain and dashed intersection points allows to project the contributions of cytoplasmic activity versus chromatin to the droplet collision-coalescence drive in distinct conditions (e.g. Control/FMN2-/-) or stages within a condition (e.g. Control NSN/Trans/SN). Source data are provided as a Source Data file.

Supplementary Fig. 8

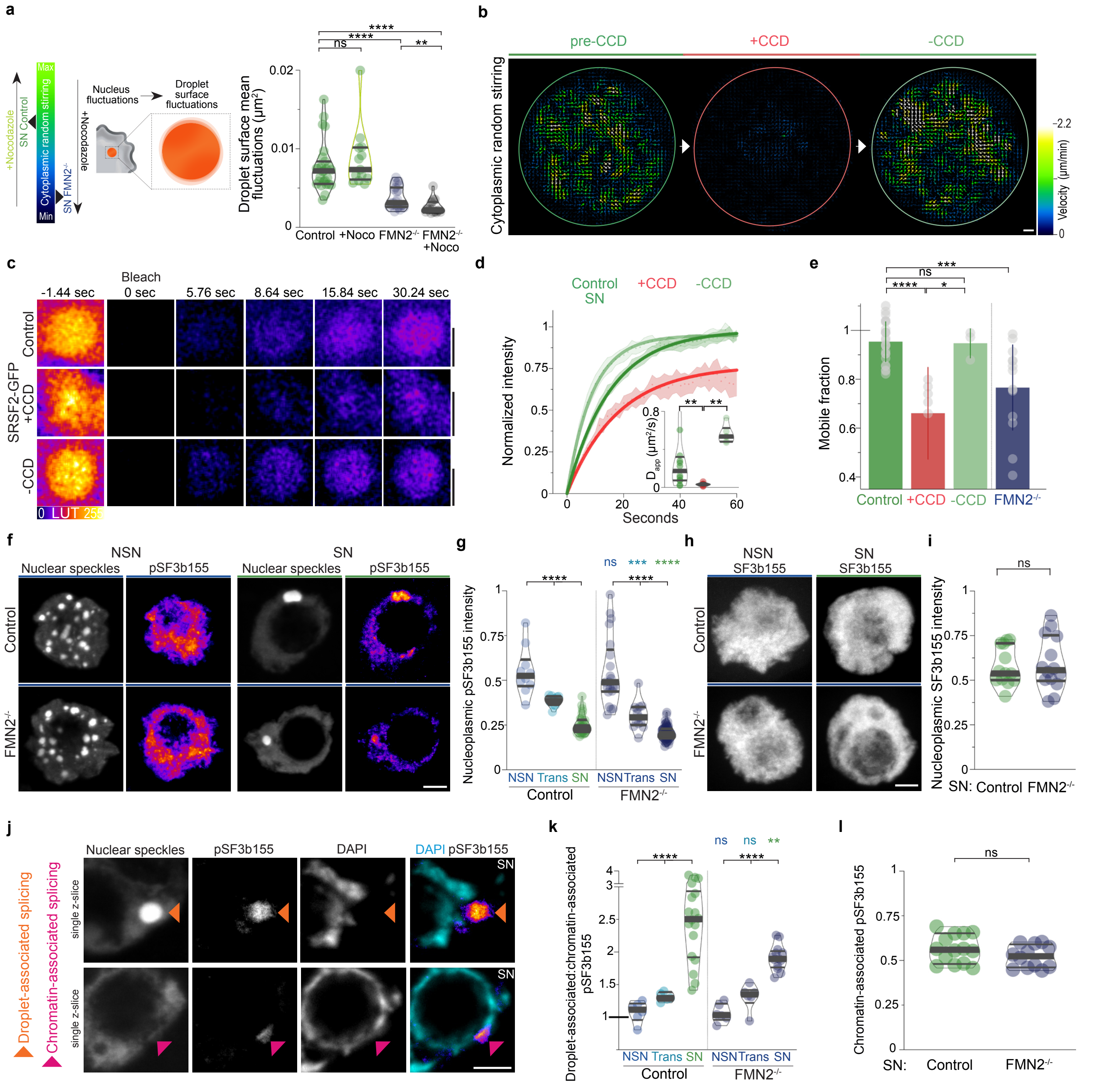

Supplementary Fig. 8 | Cytoplasmic forces enhance nuclear speckle sub-droplet scale kinetics and droplet-associated pT313-SF3b155 levels

(**a**) Cytoplasmic stirring drives droplet surface fluctuations; scheme of droplet surface fluctuations as a function of cytoplasmic stirring intensity (left); mean droplet surface fluctuations over 15 seconds (Δt=0.5s) in SN Control±Nocodazole and FMN2-/-±Nocodazole oocytes related to heatmaps in Fig.4a (right); comparably sized larger droplets were selected for analyses with a range of droplet radii from 2 to 2.7 µm; droplet number, Control=27; Nocodazole=13; FMN2-/-=23; FMN2-/-+Nocodazole=17. (**b**) Cytoplasmic stirring is restored after Cytochalasin-D washout; cytoplasmic flow vector maps generated by STICS analyses of bright-field 240 seconds-stream videos of a SN Control oocyte before Cytochalasin-D (left) that was incubated with Cytochalasin-D for 60 minutes (center; +CCD) prior to Cytochalasin-D washout for 60 minutes (right; -CCD); oocyte cortex is outlined; maps are color coded according to velocity magnitude. (**c**) Representative FRAP sequences of SRSF2-GFP droplets in SN Control oocytes, +CCD oocytes, and -CCD oocytes. (**d**) Normalized fluorescence intensity recovery curves (mean±s.e.m.) with simple exponential fits of SRSF2-GFP droplets in Control, Cytochalasin-D treated (+CCD), or Cytochalasin-D washout (-CCD) SN oocytes; insets, apparent diffusion coefficients (Dapp); droplet number, Control=12, +CCD=7, -CCD=6. (**e**) Mobile fractions of total populations for SRSF2-GFP droplets in SN Control oocytes, +CCD oocytes, -CCD oocytes, and FMN2-/- oocytes; mobile fractions correspond to recovery fractions evaluated at curve plateaus fixed at 60 seconds for all conditions; droplet number, Control=24, +CCD=6, -CCD=3, FMN2-/-=14. (**f**) Representative co-immunostainings of nuclear speckles and pT313-SF3b155 (pSF3b155) in NSN (left) and SN (right) Control or FMN2-/-oocytes (5µm z-projections); note the decrease of total nucleoplasmic pSF3b155 signal with growth progression in both Control and mutant contexts that is consistent with the drop in transcription with growth progression; also note how pSF3b155 becomes predominantly droplet-associated in both Controls and mutants during the NSN-to-SN transition. (**g**) Quantifications of global nucleoplasmic pSF3b155 intensities in Control and FMN2-/- oocytes at the three growth stages; cell number, Control NSN=10, Trans=11, SN=32, FMN2-/- NSN=21, Trans=13, SN=64. (**h**) Representative immunostainings of the non-phosphorylated form of SF3b155 showing comparable nucleoplasmic intensity levels in NSN and SN Control or FMN2-/- oocytes (35µm z-projections). (**i**) Quantifications of total nucleoplasmic SF3b155 intensity normalized by cytoplasmic noise in SN Control and FMN2-/- oocytes; cell number, Control=13, FMN2-/-=14. (**j**) Representative single z-sections of co-immunostainings of pSF3b155 associated with nuclear speckles (orange arrowhead) or with DNA (fuchsia arrowhead) in SN oocytes. (**k**) Quantifications of droplet-associated to chromatin-associated pSF3b155 signal ratios in Control and FMN2-/-oocytes at the three growth stages; cell number, Control NSN=6, Trans=5, SN=18, FMN2-/- NSN=6, Trans=6, SN=13. (**l**) Quantifications of chromatin-associated pSF3b155 in 14 Control and 12 FMN2-/- SN oocytes. Violin plots with median±quartiles (**a, d, g, i, k-l**); error represents mean±s.d. (**e**); P values derived from two-tailed Mann-Whitney U-Tests (**a, d, e, i, k-l**) and from Kruskal-Wallis Tests (**g**), ns, not significant, P>0.3307, *P=0.0238, **P<0.0083, ***P<0.0005, ****P<0.0001; scale bars, (**c**) 3µm and (**b, f, h, j**) 5µm. Source data are provided as a Source Data file.

Supplementary Fig. 9

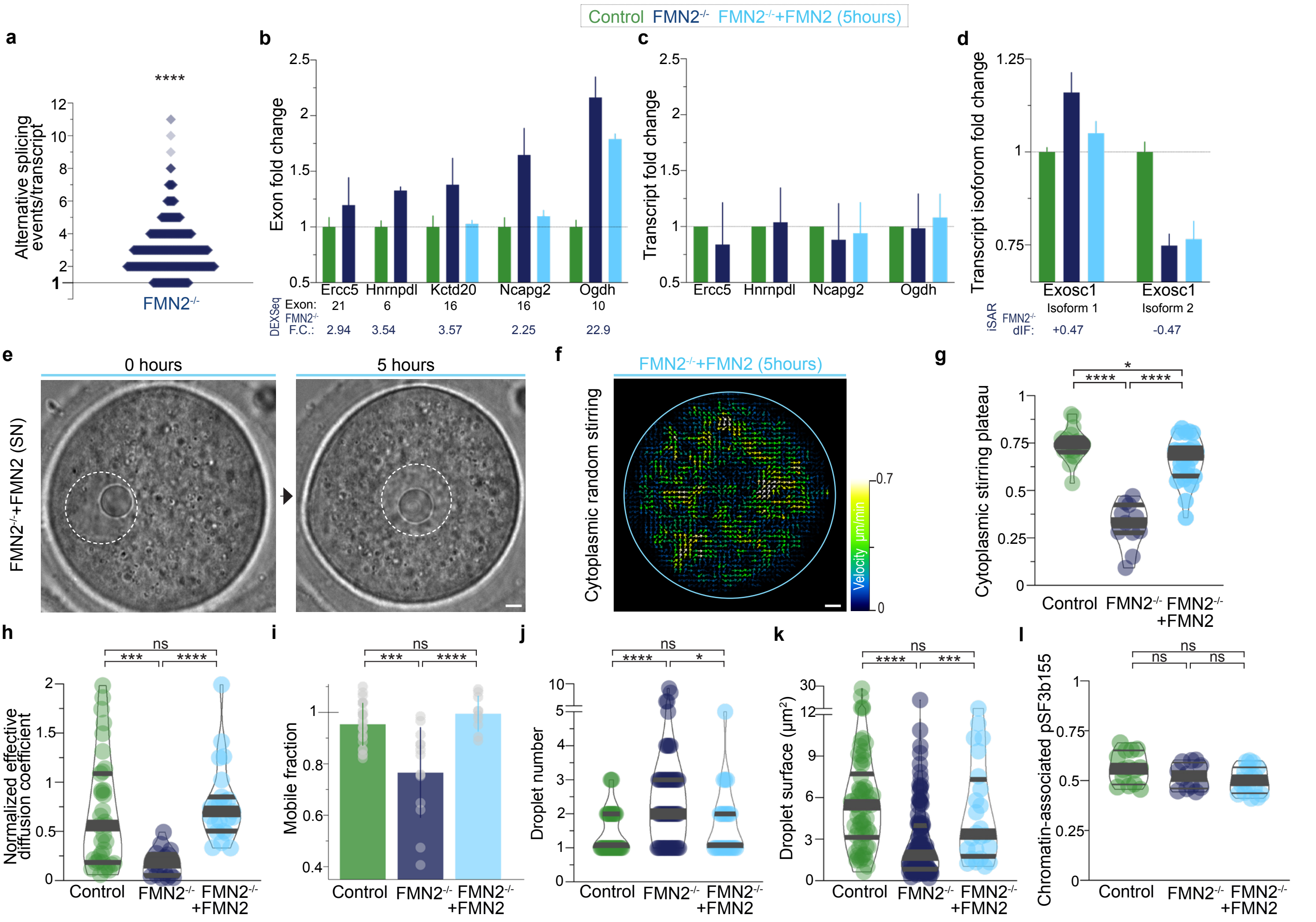

Supplementary Fig. 9 | Cytoplasmic force disruption alters mRNA splicing; restoring cytoplasmic forces in mutant SN oocytes fully rescues droplet number, size, and multiscale dynamics along with a partial reversal of mRNA splicing pattern alterations within 5 hours

(**a**) Multiple alternative splicing events occur per transcript in FMN2-/- oocytes with a median of 3; each plotted dot corresponds to one or more alternative splicing events detected in a single individual transcript for a total of 1259 transcripts. (**b-c**) RT-qPCR validation of FMN2-/- differential exon usage in distinct genes obtained bioinformatically with DEXSeq (Supplementary Data 1) and partial reversal of tendencies after expression of FMN2 in FMN2-/- oocytes for 5 hours; exon quantities are in (**b**) and associated transcript quantities are in (**c**); results are averaged from three independent experiments (mean±s.e.m.) with heterozygous FMN2+/- as Controls; FMN2-/- exon usage tendencies were confirmed (compare with DEXSeq fold change values below); note that exon numbers shown were defined by DEXSeq. (**d**) RT-qPCR validation of FMN2-/- alternative splicing detected bioinformatically with IsoformSwitchAnalyzeR (Supplementary Data 2) and partial reversal of tendencies after expression of FMN2 in FMN2-/- oocytes for 5 hours; results are averaged from three independent experiments (mean±s.e.m.) with heterozygous FMN2+/- as Controls; FMN2-/- alternative splicing tendencies were confirmed in Exosc1 transcript isoforms (compare with dIF values below). (**e**) Time-lapse of an FMN2-/- SN oocyte microinjected with FMN2 cRNA showing the repositioning of the nucleus within 5 hours. (**f**) Cytoplasmic stirring in FMN2-/- SN oocytes is restored after FMN2 expression; cytoplasmic flow vector map generated by STICS analyses of a bright-field 120 seconds-stream video of a SN FMN2-/- oocyte 5 hours after microinjection with FMN2 cRNA; oocyte cortex is outlined; maps are color coded according to velocity magnitude. (**g**) Cytoplasmic stirring intensity plateaus in SN Control, FMN2-/-, and FMN2-/-+FMN2 (5hours) oocytes obtained with image correlation analyses of cytoplasmic stirring in live oocytes for 100 seconds; cell number, Control=18, FMN2-/-=11, FMN2-/-+FMN2=24. (**h**) Normalized effective diffusion coefficients of SRSF2-GFP droplets in SN Control, FMN2-/-, and FMN2-/-+FMN2 (5hours) oocytes; effective diffusion coefficients were normalized by droplet size (D_{eff} in $\mu m^2 s^{-1}$ x 3/2 πr in µm); droplet number, Control=35, FMN2-/- =18, FMN2-/-+FMN2=23. (**i**) Mobile fractions of total populations for SRSF2-GFP droplets in SN Control, FMN2-/-, and FMN2-/-+FMN2 (5hours) oocytes; mobile fractions correspond to recovery fractions evaluated at curve plateaus fixed at 60 seconds for all conditions; droplet number, Control=24, FMN2-/-=14, FMN2-/-+FMN2=10; related to Fig.4b-c. (**j-k**) Quantifications of nuclear speckle droplet number (**m**) and surface (**n**) in nuclei of fully grown SN Control, FMN2-/-, and FMN2-/-+FMN2 (5hours) oocytes; speckles counted in 41 Control, 52 FMN2-/-, and 24 FMN2-/-+FMN2 cells, measured 62 Control, 112 FMN2-/- , and 24 FMN2-/-+FMN2 droplets. (**l**) Quantifications of chromatin-associated pSF3b155 signal in 14 Control, 12 FMN2-/- and 17 FMN2-/-+FMN2 SN oocytes. Violin plots with median±quartiles (**g-l**); P values derived from Wilcoxon Signed-Rank Test against a hypothetical median of 1(**a**) and two-tailed Mann-Whitney U-Tests (**g-l**), ns, not significant, P>0.055, *P<0.0233, ***P<0.0009, ****P<0.0001; scale bars, 5µm. Source data are provided as a Source Data file.

Supplementary Fig. 10

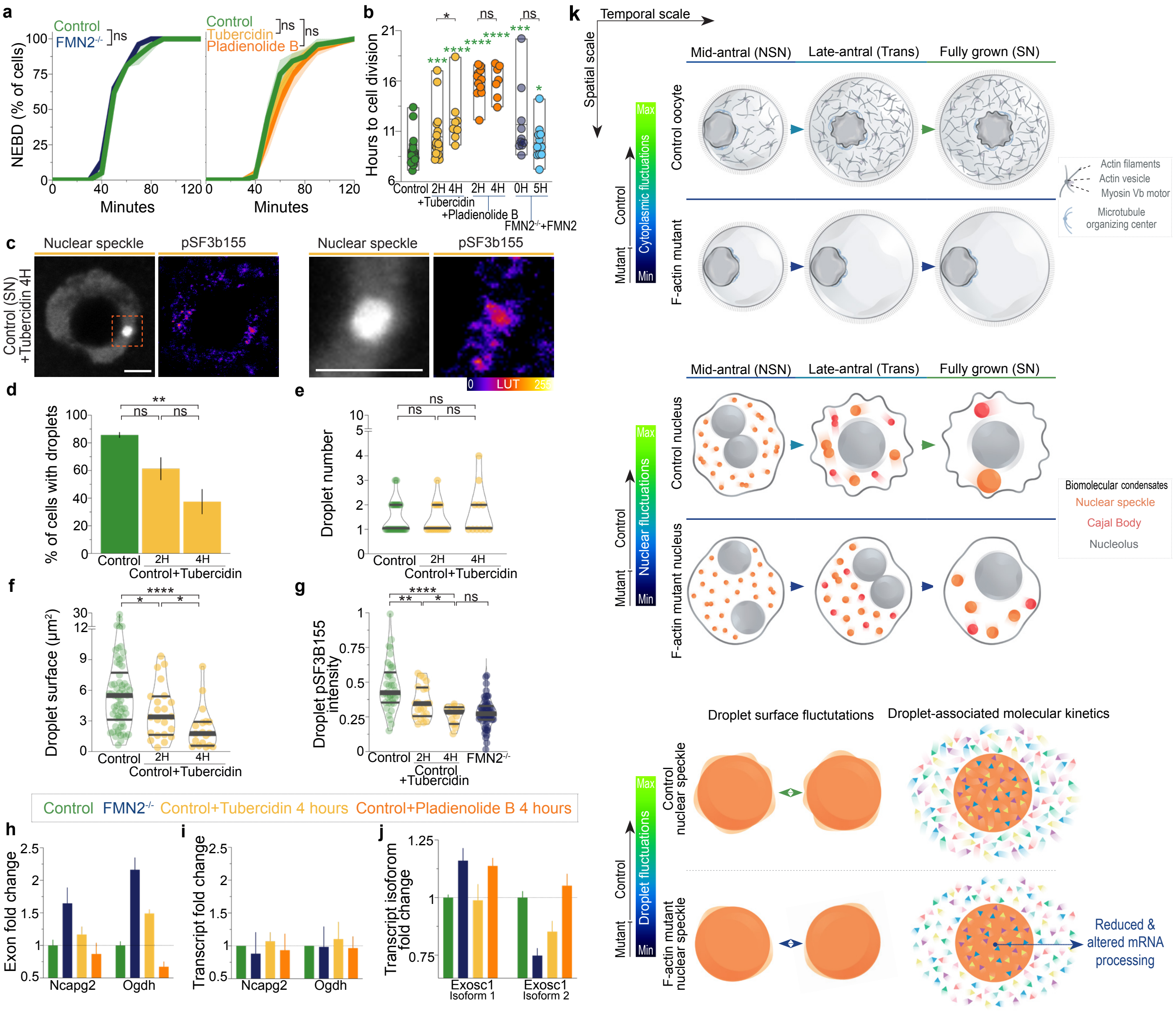

Supplementary Fig. 10 | Consequences of splicing modulation in SN oocytes; model with cytoplasmic stirring forces as scale-crossing organizers of nuclear condensates in growing oocytes

(**a**) Timing of nuclear envelope breakdown (NEBD) in percent of cells for Control and FMN2-/- SN oocytes (left) or for Control, Control+Tubercidin (2-4 hours), and Control+Pladieonolide B (2-4 hours) oocytes (right); SN oocytes were washed 6 times in fresh M2+BSA medium to remove traces of Milrinone and splicing modulators before tracking NEBD every 10 minutes for 2 hours; cell number, Control=20, FMN2-/-=53 , Control=60, Control +Tubercidin 2-4H=78, Control+Pladieonolide B 2H-4H=70. (**b**) Time necessary in hours to complete the meiotic division for oocytes that did divide after treatments with splicing modulators (2-4 hours of Tubercidin or Pladienolide B) or FMN2-rescues (0-5 hour incubation after microinjection) at the SN stage; cells were filmed every 3 minutes up to 21 hours; NEBD defined the start point and polar body extrusion defined the timing of cell division; cell number, Control=27, Control+Tubercidin 2H=18, 4H=8, Control+Pladieonolide B 2H=13, 4H=7, FMN2-/-+FMN2 0H=10, 5H=12. (**c**) Representative co-immunostainings of nuclear speckles and pSF3b155 in SN Control oocytes treated for 4 hours with Tubercidin (5µm z-projections); dashed orange box indicates the magnification shown on the right. (**d**) Proportions of SN Control oocytes±Tubercidin (2 to 4 hours) that contain nuclear speckle droplets in their nucleus; cell number, Control=54, Control+Tubericidin 2H=27,4H=16. (**e**) Quantifications of nuclear speckle droplet number in SN Control oocytes ±Tubercidin (2 to 4 hours); cell number, Control=41, Control+Tubericidin 2H=16,4H=11. (**f**) Quantifications of nuclear speckle droplet surface in SN Control oocytes±Tubercidin (2 to 4 hours); droplet number, Control=62, Control+Tubericidin 2H=22,4H=18. (**g**) Quantifications of single nuclear speckle droplet-associated pSF3b155 intensities in SN Control oocytes±Tubercidin (2 to 4 hours); droplet number, Control=38, Control+Tubericidin 2H=21,4H=15. (**h-j**) Assessment of alternative splicing tendencies in SN oocytes using RT-qPCR of Control+Tubercidin (4 hours) and Control +Pladieonolide B (4 hours) cells relative to Control and FMN2-/- cells; exon quantities are in (**h**) and associated transcript quantities are in (**i**); alternative splicing tendencies were assessed for Exosc1 transcript isoforms (**j**); results are averaged from three independent experiments (mean ±s.e.m.); related to Supplementary Fig.9b-d. Floating bars with mean±range (**b**), histogram with mean±s.e.m. (**d**), violin plots with median±quartiles (**e-g**); P values derived from P values derived from two-tailed Wilcoxon matched-pairs signed rank tests (**a**) and two-tailed Mann-Whitney U-Tests (**b, d-g**), ns, not significant, P>0.0577, *P<0.0278,**P<0.0088,***P<0.0005, ****P<0.0001; scale bars, 5µm. Source data are provided as a Source Data file.(**k**) Mouse oocyte growth model with cytoplasmic random stirring forces as multiscale and functional reorganizers of nuclear droplets. At the cell scale (top), the intensification of cytoplasmic stirring nonspecifically transports the nucleus over tens of microns while rapidly agitating it[4,14,15]. At the nucleus scale (center), the intensification of cytoplasmic stirring orchestrates reorganization of nuclear compartments, by accelerating diffusive dynamics and collision-coalescence of liquid biomolecular condensates. At the droplet scale (bottom), cytoplasmic stirring drives nuclear droplet surface fluctuations and boosts droplet-associated molecular kinetics, enhancing condensate-associated biomolecular reactions and promoting meiotic success. Inversely, cells with diminished cytoplasmic stirring due to cytoskeletal defects present organizational flaws associated with cell-, nucleus-, and droplet-scale dynamics. Weak cytoplasmic stirring like in F-actin mutants is insufficient to displace the nucleus or drive proper multiscale reorganization of nuclear liquid condensates. This multiscale disorganization leads to droplet-associated alterations in the processing of mRNA and major defects in subsequent oocyte development that drives fertility. Thus, cells can deploy cytoplasmic forces to functionally refashion liquid compartments in membrane-bound organelles like the nucleus for developmental success.

Supplementary Movie 1-7 Legends

Movie 1 | Live-imaging of cytoplasmic random stirring in growing Control and fully-grown Control or F-actin mutant oocytes
Time-lapse stream-mode imaging in bright-field of a Control growing (NSN from a mid-antral follicle; left) oocyte and fully-grown Control and FMN2$^{-/-}$ mouse oocytes (SN; center and right) showing the increase in short-timescale cytoplasmic stirring with growth and disrupted activity in the fully-grown F-actin mutant; note the peripheral nuclei in Control NSN and FMN2$^{-/-}$cells and the presence of 2 visible nucleoli in the NSN nucleus. See Fig.1 and Supplementary Fig.1. Time in mm:ss; scale bar, 5µm.

Movie 2 | Live-imaging of SRSF2-GFP droplet coalescence in the fully-grown oocyte nucleus
Time-lapse stream-mode imaging of two nucleoplasmic SRSF2-GFP droplets fusing in an SN oocyte; SRSF2 is a nuclear speckle marker. See Supplementary Fig.3. Time in mm:ss; scale bar, 5µm.

Movie 3 | Live-imaging of local nuclear SRSF2-GFP droplet displacements and collision-coalescence driven by cytoplasmic stirring in growing oocytes
Time-lapse stream-mode imaging of nucleoplasmic SRSF2-GFP droplets physically pushed by cytoplasm-based random kicks of the nuclear membrane in NSN and SN oocytes; droplet collision-coalescence is instigated by the cytoplasm-based forces that locally displace the NSN droplet; note the more rapid diffusive dynamics of the SN droplet. See Fig.2 and Supplementary Fig.5. Time in mm:ss; scale bar, 5µm.

Movie 4 | Live-imaging of global nuclear SRSF2-GFP droplet dynamics in growing oocytes with Control or modulated cytoplasmic forces
Time-lapse stream-mode imaging of global SRSF2-GFP droplet diffusive dynamics in nucleoplasms of NSN (1st row) and SN (2nd row) oocytes with Control cytoplasmic forces (1st column), amplified forces (+Nocodazole; 2nd column), obstructed forces (+Taxol; 3rd column), and disrupted forces (FMN2$^{-/-}$; 4th column). See Fig.2 and Supplementary Fig.5. Time in mm:ss; scale bar, 5µm.

Movie 5 | Live-imaging of nuclear SRSF2-GFP droplets with random large-scale displacements and collision-coalescence in growing oocytes
Time-lapse images (50 µm z-projections) of global nucleoplasmic SRSF2-GFP droplet dynamics in Trans and SN oocytes on longer timescales; note random large-scale displacements of droplets and occasional collision-coalescence. See Fig.2 and Supplementary Fig.6. Time in hh:mm; scale bar, 5µm.

Movie 6 | Live-imaging of nuclear SRSF2-GFP droplet coalescence dynamics in growing oocytes with Control or modulated cytoplasmic forces
Time-lapse images (40 µm z-projections) of nucleoplasmic SRSF2-GFP droplet dynamics showing collision-coalescence evolution on longer timescales in NSN oocytes with Control cytoplasmic forces (1st column), amplified forces (+Nocodazole; 2nd column), obstructed forces (+Taxol; 3rd column), and disrupted forces (FMN2$^{-/-}$; 4th column); note that starting point droplet numbers for each condition vary slightly. See Fig.2 and Supplementary Fig.6. Time in hh:mm; scale bar, 5µm.

Movie 7 | 3D-simulations of nuclear SRSF2 droplet collision-coalescence evolution from NSN-like to SN-like states relative to cytoplasmic force intensity
Time-lapse images of 3D-simulations on hour to day timescales showing the evolution of nuclear SRSF2-like droplet collision-coalescence relative to varying intensities of cytoplasmic stirring activity;

NSN-to-SN-like simulation regime with first 12 hours of simulations performed with NSN-like parameters before a nuclear obstacle and cytoplasmic activity switch that occurs at 12 hours whereby 40 % of chromatin-like obstacles surround the nucleolus and cytoplasmic activity nearly doubles to mimic the transition into the SN-like condition (physiologically marked by chromatin condensation and cytoplasmic force intensification). $FMN2^{-/-}$-like cytoplasmic forces (0.11-0.19; 1st column), Control-like forces (0.55-1; 2nd column), ~two-fold amplified forces (1.05-1.89; Control+Nocodazole-like; 3rd column), and ~four-fold amplified forces (2.32-4.18; 4th column). SRSF2 droplets are in orange and the nucleolus is in light grey placed in a dark grey spherical nucleus; chromatin-like obstacles are invisible. See Fig.3 and Supplementary Fig.7. Time in hh:mm; scale bar, 5μm.

Supplementary Data 1-5 Legends

Supplementary Data 1 | mRNA exon usage table ($FMN2^{+/-}$ vs. $FMN2^{-/-}$ SN oocytes)
Sheet 1, DEXSeq output with P_{adj}<0.05. Sheet 2, Illustration summarizing primer pair design for RT-qPCR experiments. See Fig. 4 and Supplementary Fig.9.

Supplementary Data 2 | mRNA isoform usage table ($FMN2^{+/-}$ vs. $FMN2^{-/-}$ SN oocytes)
Sheet 1, IsoformSwitchAnalyzeR (iSAR) output with P_{adj}<0.05. Sheet 2, detected alternative splicing events. Sheet 3, transcript ID and splicing coordinates. Sheet 4, Predicted isoform switch consequences. Sheet 5, isoforms selected for RT-qPCR validation and illustration of primer pair design. See Fig. 4 and Supplementary Fig.9.

Supplementary Data 3 | Enrichment and spatial correlation analyses of altered splicing sites relative to SRSF1 and SRSF2 binding sites
Sheet 1-2, Enrichment analyses of SRSF1 and SRSF2 binding sites[16] in $FMN2^{-/-}$ genes associated with altered splicing relative to all genomic transcripts; statistical tests are displayed in sheet 2. Sheet 3-4, Spatial correlation analyses using the GenometriCorr package[17] comparing $FMN2^{-/-}$ altered splicing sites with binding sites of SRSF1, SRSF2, MBNL3, or YY1([16,18,19]); *in silico* controls correspond to the 50 first (Prom50) or last (Term50) nucleotides of all RefSeqNCBI transcripts; see Fig.4.

Supplementary Data 4 | Functional enrichment of $FMN2^{-/-}$ genes with mRNA processing alterations
Enrichr Gene Ontology – Biological Process output for the list of $FMN2^{-/-}$ genes affected by mRNA processing defects revealed by DEXSeq and iSAR.

Supplementary Data 5 | $FMN2^{-/-}$ genes with mRNA processing alterations compared to translated ones during the first meiotic division
$FMN2^{-/-}$ genes with altered mRNA processing compared to translational status of transcripts during the first meiotic division[20]. Sheet 1, list of translated, activated (engaged in translation), and repressed (degraded) transcripts during the first meiotic division; GeneID MGI-conversions are next to each list. Sheet 2, Comparative analysis showing that 53% of $FMN2^{-/-}$ genes with altered mRNA processing (DEXSeq and iSAR lists) are translated or engaged in translation during the first meiotic division.

Supplementary references

1. Tan, J. H. *et al.* Chromatin configurations in the germinal vesicle of mammalian oocytes. *Mol. Hum. Reprod.* **15**, 1–9 (2009).
2. Bonnet-Garnier, A. *et al.* Genome organization and epigenetic marks in mouse germinal vesicle oocytes. *Int. J. Dev. Biol.* **56**, 877–887 (2012).
3. Bogolyubova, I. & Bogolyubov, D. Heterochromatin Morphodynamics in Late Oogenesis and Early Embryogenesis of Mammals. *Cells* **9**, 1–30 (2020).
4. Almonacid, M. *et al.* Active Fluctuations of the Nuclear Envelope Shape the Transcriptional Dynamics in Oocytes. *Dev. Cell* **51**, 145–157 (2019).
5. Roeles, J. & Tsiavaliaris, G. Actin-microtubule interplay coordinates spindle assembly in human oocytes. *Nat. Commun.* **10**, 1–10 (2019).
6. Brunet, S. & Maro, B. Germinal vesicle position and meiotic maturation in mouse oocyte. *Reproduction* **133**, 1069–1072 (2007).
7. Sawyer, I. A., Sturgill, D. & Dundr, M. Membraneless nuclear organelles and the search for phases within phases. *Wiley Interdiscip. Rev. RNA* **10**, 1–20 (2019).
8. Sabari, B. R., Dall'Agnese, A. & Young, R. A. Biomolecular Condensates in the Nucleus. *Trends Biochem. Sci.* 1–17 (2020). doi:10.1016/j.tibs.2020.06.007
9. Lafontaine, D. L. J., Riback, J. A., Bascetin, R. & Brangwynne, C. P. The nucleolus as a multiphase liquid condensate. *Nat. Rev. Mol. Cell Biol.* (2020).
10. Ribbeck, K. & Görlich, D. The permeability barrier of nuclear pore complexes appears to operate via hydrophobic exclusion. *EMBO J.* **21**, 2664–2671 (2002).
11. Strom, A. R. *et al.* Phase separation drives heterochromatin domain formation. *Nature* **547**, 241–245 (2017).
12. Kroschwald, S., Maharana, S. & Simon, A. Hexanediol: a chemical probe to investigate the material properties of membrane-less compartments. *Matters* 201702000010 (2017).
13. Jao, C. Y. & Salic, A. Exploring RNA transcription and turnover in vivo by using click chemistry. *Proc. Natl. Acad. Sci. U. S. A.* **105**, 15779–15784 (2008).
14. Almonacid, M. *et al.* Active diffusion positions the nucleus in mouse oocytes. *Nat. Cell Biol.* **17**, 470–479 (2015).
15. Colin, A. *et al.* Active diffusion in oocytes nonspecifically centers large objects during prophase I and meiosis I. *J. Cell Biol.* **219**, e201908195 (2020).
16. Pandit, S. *et al.* Genome-wide Analysis Reveals SR Protein Cooperation and Competition in Regulated Splicing. *Mol. Cell* **50**, 223–235 (2013).
17. Favorov, A. *et al.* Exploring Massive , Genome Scale Datasets with the GenometriCorr Package. *PLoS Comput. Biol.* **8**, e1002529 (2012).
18. Poulos, M. G. *et al.* Progressive impairment of muscle regeneration in muscleblind-like 3 isoform knockout mice. *Hum. Mol. Genet.* **22**, 3547–3558 (2013).
19. Sigova, A. A. *et al.* Transcription factor trapping by RNA in gene regulatory elements. *Science* **350**, 978–982 (2015).
20. Chen, J. *et al.* Genome-wide analysis of translation reveals a critical role for deleted in azoospermia-like (Dazl) at the oocyte-to-zygote transition. *Genes Dev.* **25**, 755–766 (2011).
