# Supplementary material for: Cytoplasmic forces functionally reorganize nuclear condensates in oocytes
Source: Nat Commun. 2022 Aug 29;13:5070. doi: 10.1038/s41467-022-32675-5 (PMC9424315; doi:10.1038/s41467-022-32675-5)
Supplement: Supplementary file 2 — Description of Additional Supplementary Files [file 41467_2022_32675_MOESM2_ESM.pdf]

Description of Additional Supplementary Files

Supplementary Data files 1-5

Supplementary Data 1 | mRNA exon usage table (FMN2$^{+/-}$ vs. FMN2$^{-/-}$ SN oocytes)
Sheet 1, DEXSeq output with P_{adj}<0.05. Sheet 2, Illustration summarizing primer pair design for RT-qPCR experiments. See Fig. 4 and Supplementary Fig.9.

Supplementary Data 2 | mRNA isoform usage table (FMN2$^{+/-}$ vs. FMN2$^{-/-}$ SN oocytes)
Sheet 1, IsoformSwitchAnalyzeR (iSAR) output with P_{adj}<0.05. Sheet 2, detected alternative splicing events. Sheet 3, transcript ID and splicing coordinates. Sheet 4, Predicted isoform switch consequences. Sheet 5, isoforms selected for RT-qPCR validation and illustration of primer pair design. See Fig. 4 and Supplementary Fig.9.

Supplementary Data 3 | Enrichment and spatial correlation analyses of altered splicing sites relative to SRSF1 and SRSF2 binding sites
Sheet 1-2, Enrichment analyses of SRSF1 and SRSF2 binding sites[16] in FMN2$^{-/-}$ genes associated with altered splicing relative to all genomic transcripts; statistical tests are displayed in sheet 2. Sheet 3-4, Spatial correlation analyses using the GenometriCorr package[17] comparing FMN2$^{-/-}$ altered splicing sites with binding sites of SRSF1, SRSF2, MBNL3, or YY1([16,18,19]); *in silico* controls correspond to the 50 first (Prom50) or last (Term50) nucleotides of all RefSeqNCBI transcripts; see Fig.4.

Supplementary Data 4 | Functional enrichment of FMN2$^{-/-}$ genes with mRNA processing alterations
Enrichr Gene Ontology – Biological Process output for the list of FMN2$^{-/-}$ genes affected by mRNA processing defects revealed by DEXSeq and iSAR.

Supplementary Data 5 | FMN2$^{-/-}$ genes with mRNA processing alterations compared to translated ones during the first meiotic division
FMN2$^{-/-}$ genes with altered mRNA processing compared to translational status of transcripts during the first meiotic division[20]. Sheet 1, list of translated, activated (engaged in translation), and repressed (degraded) transcripts during the first meiotic division; GeneID MGI-conversions are next to each list. Sheet 2, Comparative analysis showing that 53% of FMN2$^{-/-}$ genes with altered mRNA processing (DEXSeq and iSAR lists) are translated or engaged in translation during the first meiotic division.

Supplementary Movie 1-7

Movie 1 | Live-imaging of cytoplasmic random stirring in growing Control and fully-grown Control or F-actin mutant oocytes

Time-lapse stream-mode imaging in bright-field of a Control growing (NSN from a mid-antral follicle; left) oocyte and fully-grown Control and FMN2-/- mouse oocytes (SN; center and right) showing the increase in short-timescale cytoplasmic stirring with growth and disrupted activity in the fully-grown F-actin mutant; note the peripheral nuclei in Control NSN and FMN2-/-cells and the presence of 2 visible nucleoli in the NSN nucleus. See Fig.1 and Supplementary Fig.1. Time in mm:ss; scale bar, 5µm.

Movie 2 | Live-imaging of SRSF2-GFP droplet coalescence in the fully-grown oocyte nucleus

Time-lapse stream-mode imaging of two nucleoplasmic SRSF2-GFP droplets fusing in an SN oocyte; SRSF2 is a nuclear speckle marker. See Supplementary Fig.3. Time in mm:ss; scale bar, 5µm.

Movie 3 | Live-imaging of local nuclear SRSF2-GFP droplet displacements and collision-coalescence driven by cytoplasmic stirring in growing oocytes

Time-lapse stream-mode imaging of nucleoplasmic SRSF2-GFP droplets physically pushed by cytoplasm-based random kicks of the nuclear membrane in NSN and SN oocytes; droplet collision-coalescence is instigated by the cytoplasm-based forces that locally displace the NSN droplet; note the more rapid diffusive dynamics of the SN droplet. See Fig.2 and Supplementary Fig.5. Time in mm:ss; scale bar, 5µm.

Movie 4 | Live-imaging of global nuclear SRSF2-GFP droplet dynamics in growing oocytes with Control or modulated cytoplasmic forces

Time-lapse stream-mode imaging of global SRSF2-GFP droplet diffusive dynamics in nucleoplasms of NSN (1st row) and SN (2nd row) oocytes with Control cytoplasmic forces (1st column), amplified forces (+Nocodazole; 2nd

column), obstructed forces (+Taxol; 3rd column), and disrupted forces (FMN2$^{-/-}$; 4th column). See Fig.2 and Supplementary Fig.5. Time in mm:ss; scale bar, 5µm.

Movie 5 | Live-imaging of nuclear SRSF2-GFP droplets with random large-scale displacements and collision-coalescence in growing oocytes

Time-lapse images (50 µm z-projections) of global nucleoplasmic SRSF2-GFP droplet dynamics in Trans and SN oocytes on longer timescales; note random large-scale displacements of droplets and occasional collision-coalescence. See Fig.2 and Supplementary Fig.6. Time in hh:mm; scale bar, 5µm.

Movie 6 | Live-imaging of nuclear SRSF2-GFP droplet coalescence dynamics in growing oocytes with Control or modulated cytoplasmic forces

Time-lapse images (40 µm z-projections) of nucleoplasmic SRSF2-GFP droplet dynamics showing collision-coalescence evolution on longer timescales in NSN oocytes with Control cytoplasmic forces (1st column), amplified forces (+Nocodazole; 2nd column), obstructed forces (+Taxol; 3rd column), and disrupted forces (FMN2$^{-/-}$; 4th column); note that starting point droplet numbers for each condition vary slightly. See Fig.2 and Supplementary Fig.6. Time in hh:mm; scale bar, 5µm.

Movie 7 | 3D-simulations of nuclear SRSF2 droplet collision-coalescence evolution from NSN-like to SN-like states relative to cytoplasmic force intensity

Time-lapse images of 3D-simulations on hour to day timescales showing the evolution of nuclear SRSF2-like droplet collision-coalescence relative to varying intensities of cytoplasmic stirring activity; NSN-to-SN-like simulation regime with first 12 hours of simulations performed with NSN-like parameters before a nuclear obstacle and cytoplasmic activity switch that occurs at 12 hours whereby 40 % of chromatin-like obstacles surround the nucleolus and cytoplasmic activity nearly doubles to mimic the transition into the SN-like condition (physiologically marked by chromatin condensation and cytoplasmic force intensification). FMN2-/--like cytoplasmic forces (0.11-0.19; 1st column), Control-like forces (0.55-1; 2nd column), ~two-fold amplified forces (1.05-1.89; Control+Nocodazole-like; 3rd column), and ~four-fold amplified forces (2.32-

4.18; 4th column). SRSF2 droplets are in orange and the nucleolus is in light grey placed in a dark grey spherical nucleus; chromatin-like obstacles are invisible. See Fig.3 and Supplementary Fig.7. Time in hh:mm; scale bar, 5µm.
